# More metalwork removals in patients with olecranon fracture treated by tension band wiring than plate fixation-a propensity score matching analysis

**DOI:** 10.1186/s12891-021-04559-0

**Published:** 2021-08-13

**Authors:** Longhai Qiu, Yi Li, Hongbo Wu, Ruixiong Chen, Zhiwen Zhang, Xiaofeng Wang, Yuliang Huang

**Affiliations:** Department of Traumatology and Orthopaedic Surgery, Institute of Orthopaedics, Huizhou Municipal Central Hospital, Huizhou, Guangdong 516001 People’s Republic of China

**Keywords:** Olecranon fracture, Elbow instability, Complications, Mayo elbow performance score, Metalwork removal

## Abstract

**Background:**

Traditional tension band wiring and plate fixation represent the commonest methods for treating olecranon fractures. However, there is no agreement on which method provides the best outcome. The aim of this retrospective study is to compare the outcomes of tension band wiring (TBW) and plate fixation (PF) for treating displaced olecranon fractures. This is the first study to use propensity score matching analysis to compare treatment methods for olecranon fracture.

**Method:**

A total of 107 patients aged between 18 and 85 had acute isolated and displaced olecranon fractures. The patients were divided into either TBW (*n* = 49) or PF (*n* = 58) groups. To conduct propensity score matching for the treatment method (TBW versus PF), 58 patients were analyzed by logistic regression (29 patients in each group). Various demographic and treatment-related variables were examined and analyzed to determine their correlation.

**Results:**

Functional effects between two groups are similar (in terms of Mayo Elbow Performance Score (MEPS), the patients’ range of elbow motion (ROM) and forearm rotation (RFR), the time return to work (RTW)). The total adverse events rate and metalwork removal events rate are higher in TBW than that in PF. After propensity score matching analysis, similar primary treatment efficacy (indicated by MEPS> 90) in 2 groups and more primary adverse events (indicated by metalwork removal) were perceived in TBW than that in PF. Logistic regression analysis revealed that fracture type was an independent factor that affected the efficacy of a treatment *(regression coefficient = − 1.24 < 0, P = 0.03)*, indicating that fracture severity was inversely proportional to the efficacy of a treatment for olecranon fracture. Furthermore, logistic regression analysis demonstrated that the treatment method was an independent factor that affected metalwork removal of olecranon fracture *(regression coefficient 2.38 > 0, OR = 10.77, P < 0.01)*, indicating that the risk of metalwork removal in the TBW Group was 10.77 times that in the PF Group.

**Conclusion:**

When initially discussing the surgical approach with patients, physicians should fully weigh the possibility that TBW may lead to a second surgery due to the higher risk of internal fixation removal and that TBW won’t yield better functional outcomes than PF .

## Background

The olecranon is situated directly under the skin and is vulnerable to damage. Olecranon fractures account for 1% of all upper extremity fractures, with an incidence rate of 11.5 to 12 per 100,000 population annually [1, 2]. Most olecranon fractures involve the articular surface of the elbow joint, and uneven articular surfaces can cause limited elbow joints, traumatic arthritis and other complications. Surgical intervention is necessary to achieve accurate reduction and rigid fixation in cases of unstable elbow joints and osteoarthritis [3]. Despite advocates for alternative surgical techniques including intramedullary nailing and suture fixation, TBW fixation remains the standard management for simple isolated, displaced fractures of the olecranon (Mayo type 2A) [2].

Some researchers believe that plate fixation (PF) is an effective alternative, as there are few complications with this method. PF can be used for any type of olecranon fracture but it is particularly recommended for the following indications: comminuted fractures, Monteggia fracture dislocations, oblique fractures (particularly those distal to midpoint of the trochlear notch) and fractures that involve the coronoid process [4].

Though prospective studies and retrospective studies have noted comparable functional outcomes between TBW and plate fixation [5, 6], it remains unclear whether the initial higher cost of plate fixation is offset by the cost associated with the higher rate of TBW construct removal. The aim of the current study was to determine if any difference exists between TBW and plate fixation with respect to the functional outcomes (Mayo Elbow Performance Score) and adverse events (metalwork removals). To our knowledge, this is the first study to adopt propensity score matching to compare TBW and PF for the treatment of olecranon fractures.

## Methods

This is a retrospective single-center case control trial including active adult patients with a simply displaced fracture of the olecranon treated at Huizhou Central People’s Hospital (Huizhou Hospital Affiliated to Guangdong Medical University). Ethical approval was obtained from the Institutional Review Board of Guangdong Medical University, and the study conformed to the tenets of the Declaration of Helsinki. We obtained the verbal consent of the patient or his directly-related family members. The ethics committee approved this procedure because the two treatments in this study were universally applicable and the treatment itself did not require ethical approval.

### Patients

**Fig. 1:**
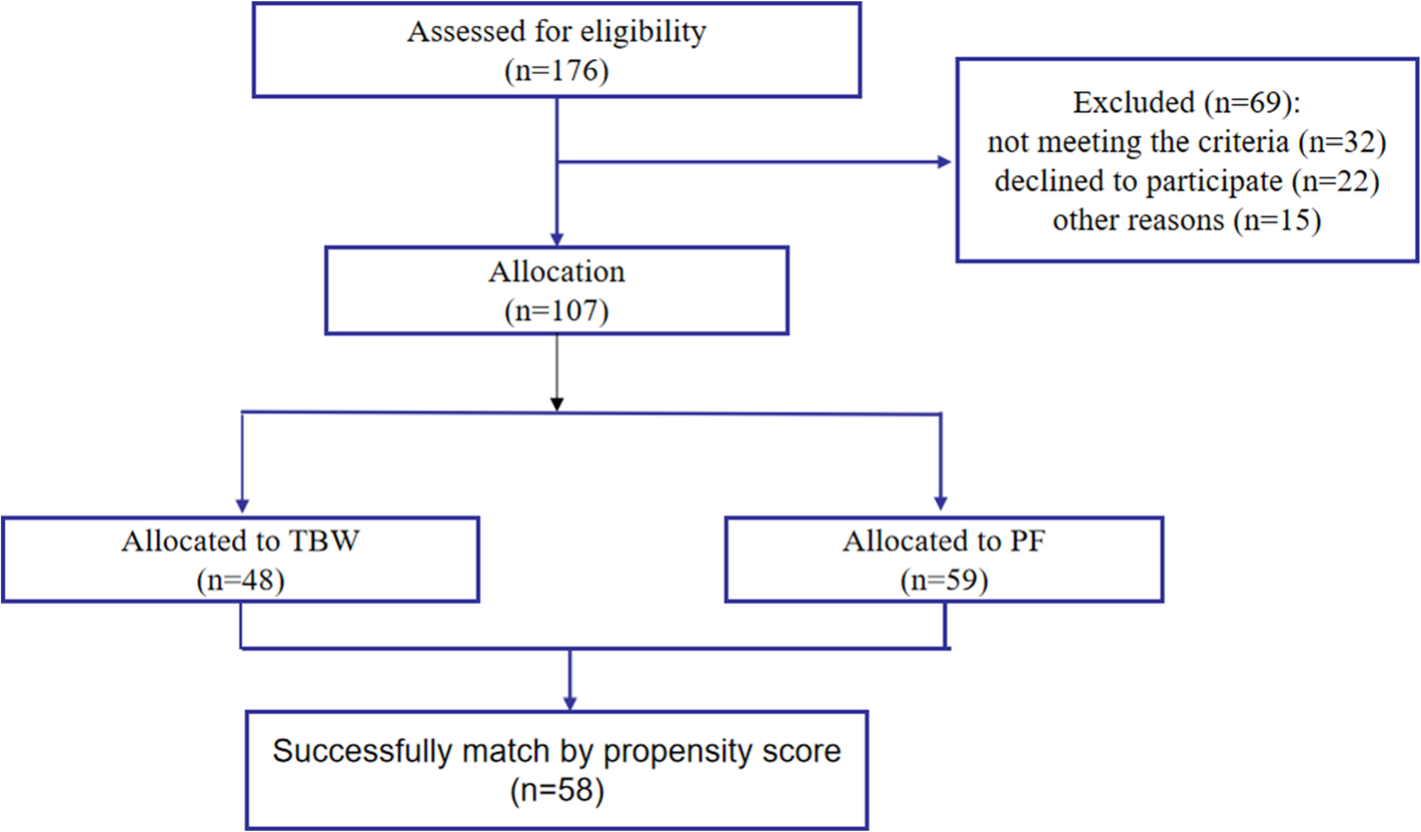
None

**Fig. 2:**
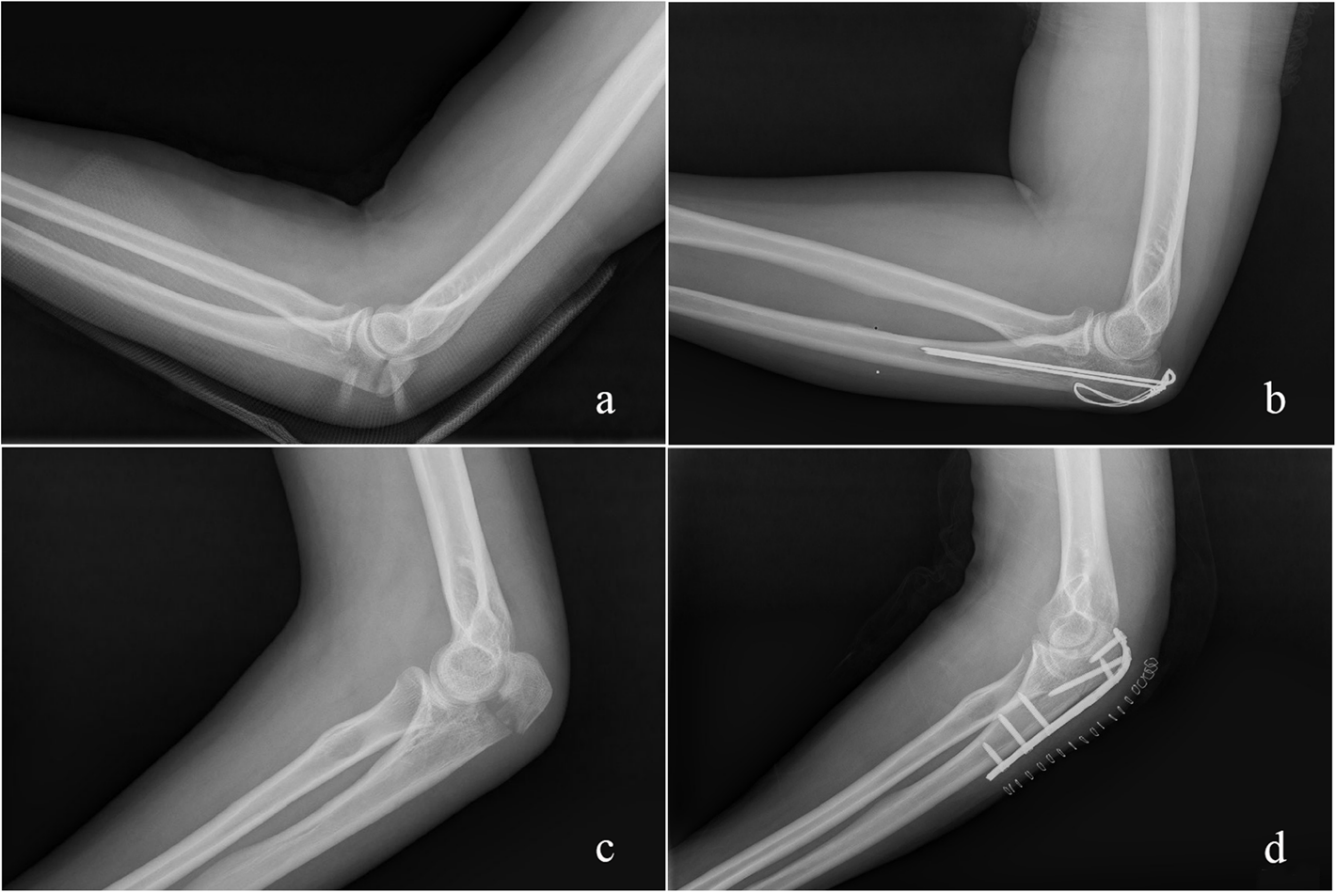
**A** Preoperative lateral radiograph of elbow demonstrating Mayo 2A olecranon fracture. **B** Postoperative radiograph after tension band wiring. **C** Prepoperative lateral radiograph of elbow demonstrating another Mayo 2A olecranon fracture. **D** Postoperative radiograph after plate fixation. This figure comes from the corespondent author’s research team

In total, 176 patients with displaced olecranon fractures (Mayo 2A and Mayo2B) were treated at our department from 2005 to 2019. Sixty nine patients were excluded from this study, and data from the remaining 107 patients were analyzed (Fig. 1). The inclusion criteria were as follows: ① aged between 18 and 85; ②displaced olecranon fractures with minimal or moderate fragmentation of olecranon; ③ olecranon fractures treated by TBW or PF (Fig. 2). The exclusion criteria were as follows: ① pregnant women/skeletal immature patients; ② patients who were unable to comply with follow-up; ③ associated fractures with radial head fracture/coronoid/distal aspects of the humerus; ④ associated ligamentous injury/dislocation/subluxation of the elbow; ⑤ previous history of fractures on the side of injury; ⑥ open olecranon fractures.

### Variables

The following clinical factors were examined retrospectively using the patients’ medical records: age; sex; career (heavy manual worker or not); mechanism of injury (fall from standing height; fall from height; motor vehicle collision; sports; fight/assault); dominant hand; side of injury; body mass index (BMI); diabetes; alcohol consumption (alcohol consumption< 21 units/wk. or alcohol consumption> 21 units/wk); smoking; American Society of Anesthesiologists (ASA) grade; associated injury (ipsilateral shoulder joint injury, ipsilateral wrist joint injury, minor head and others); type of fracture (classified by Mayo classification:2A-noncommunited fracture;2B-communited fracture); treatment methods (tension band wiring versus internal plate fixation). The fracture type was divided into 2 categories: Mayo 2A and Mayo 2B. Any disagreements regarding the radiographs and the Mayo classification were resolved by discussion within two co-first authors. The patients were divided into 2 groups based on treatment methods: the tension band wiring group (TBW Group: 48 patients) and the plate fixation group (PF Group: 57 patients). The choice of implant was made by the patients on the advice of the attending surgeons. All operations were performed by the chief surgeons with more than 5 years of independent operation experience and some of the chief surgeons are part of the authors.

### Outcome

Various functional effects were assessed as following: Mayo Elbow Performance Score (MEPS), the patients’ range of elbow motion (ROM) and forearm rotation (RFR), the time return to work (RTW). The MEPS can be divided into 4 levels: excellent (≥ 90), good (80 ~ 90), general (70 ~ 80) and poor (≥ 70). The primary functional outcome was assessed by treatment efficacy (as indicated by an excellent MEPS, a generic and upper-limb specific validated clinical score). And then the occurrences of various adverse events were recorded as following: metalwork removal, infection, revision and others (union, malunion, painful stiff elbow, persistent instability of the elbow, posttraumatic arthritis, heterotopic ossification). The primary adverse event was assessed by metalwork removal, a frequent complication predominated by prominence at the site of the proximal ulna.

### Statistical analysis

All statistical analyses were performed using SPSS software. First, the baseline data of the two groups was compared. Two-sample independent t-tests were adopted for continuous variables that followed a normal distribution, and two-sample independent rank sum tests were adopted otherwise. Chi-square test was used to compare categorical variables. Propensity score analysis was performed to minimize selection biases associated with a retrospective data analysis between the TBW Group and the PF Group. For each patient, a propensity score for the treatment method group was calculated by a logistic regression analysis of all predictive variables. Propensity score matching was calculated for all 107 patients by logistic regression analysis for all 13 factors associated with treatment. The concordance index was 0.02, indicating a strong ability to differentiate patients based on whether they were treated by PF or TBW. The Hosmer–Lemeshow statistic was nonsignificant (*P* = 0.05), indicating good calibration. The propensity scores in the PF Group ranged from 0.153 to 0.788, and the propensity scores in the TBW Group ranged from 0.153 to 0.788. The baseline variables of the 58 propensity-score-matched cases (PF versus TBW) were then evaluated as above.

Functional effects between two groups were compared by the Mann-Whitney test because the continuous data were not normally distributed. Adverse events, including various complications, were compared by the Chi-square test. Furthermore, the 58 propensity-score-matched cases were then evaluated by univariate analysis and multivariate analysis by logistic regression to identify the potential factors associated with 2 primary outcomes respectively: treatment efficacy (MEPS > 90) and primary adverse events (metalwork removal). In all analyses, two-tailed *p* values < 0.05 were considered significant.

## Results

### Comparison of variables in group TB and group PF in 107 patients before PSM and in 58 patients after PSM

The variables in Group TB and Group PF in107 patients before PSM and in 58 patients after PSM (shown in Table 1). There were differences between the TBW Group and the PF Group regarding diabetes, dominant hands and the side of injury. Propensity score matching resulted in 58 patients being matched to 29 patients in each group and there was no difference between the above 2 groups (shown in Table 1).

**Table 1:** Comparison of variables in Group TB and Group PF in 107 patients before PSM and in 58 patients after PSM

		Group (before PSM)		χ2/Z	*P*	Group (after PSM)		χ2 /Z	*P*
		TBW	PF			TBW	PF		
Sex	Male	24 (22.43)	34 (31.78)	0.62	0.43	14 (24.14)	18 (31.03)	1.12	0.21
	Female	24 (22.43)	25 (23.36)			15 (25.86)	11 (18.97)		
Age		35 (22,59.8)	35 (22,59.8)	−1.24	0.22	33 (22,51)	38 (30,55.5)	−1.39	0.17
Occupation	Heavy worker	13 (12.15)	16 (14.95)	0	0.99	20 (34.48)	20 (34.48)	0	> 0.05
	No heavy worker	35 (32.71)	43 (40.19)			9 (15.52)	9 (15.52)		
Associated injury	None	22 (20.56)	33 (30.84)	5	0.29	16 (27.59)	13 (22.41)	3.27	0.55
	Ipsilateral shoulder joint injury	5 (4.67)	9 (8.41)			3 (5.17)	5 (8.62)		
	Ipsilateral wrist joint injury	7 (6.54)	2 (1.87)			5 (8.62)	2 (3.45)		
	Minor head injury	8 (7.48)	9 (8.41)			3 (5.17)	6 (10.34)		
	Others	6 (5.61)	6 (5.61)			2 (3.45)	3 (5.17)		
Mechanism of injury	Fall from standing height	19 (17.76)	22 (20.56)	2.06	0.73	14 (24.14)	10 (17.24)	2.08	0.75
	Fall from height	6 (5.61)	12 (11.21)			3 (5.17)	5 (8.62)		
	Motor vehicle collision	11 (10.28)	15 (14.02)			6 (10.34)	9 (15.52)		
	Sports	7 (6.54)	5 (4.67)			4 (6.9)	3 (5.17)		
	Fight/assault	5 (4.67)	6 (5.61)			2 (3.45)	2 (3.45)		
Diabetes	No	39 (36.45)	56 (52.34)	4.96	0.03	27 (46.55)	29 (50)	2.07	0.49
	Yes	9 (8.41)	3 (2.80)			2 (3.45)	0 (0)		
Dominant hand	Left hand	0 (0)	6 (5.61)	5.17	0.03	0 (0)	0 (0)	–	–
	Right hand	48 (44.86)	53 (49.53)			29 (50)	29 (50)		
Side of injury	Left hand	24 (22.43)	41 (38.32)	4.22	0.048	19 (32.76)	17 (29.31)	0.29	0.79
	Right hand	24 (22.43)	18 (16.82)			10 (17.24)	12 (20.69)		
ASA grade	I	34 (31.78)	41 (38.32)	−0.14	0.89	1 (1,1.5)	1 (1,1)	−0.23	0.97
	II	10 (9.35)	13 (12.15)						
	III	4 (3.74)	5 (4.67)						
Alcohol consumption	< 21 units/wk	42 (39.25)	55 (51.40)	1.02	0.34	28 (48.28)	26 (44.83)	1.07	0.61
	> 21 units/wk	6 (5.61)	4 (3.74)			1 (1.72)	3 (5.17)		
Smoking	No	34 (31.78)	41 (38.32)	0.023	1.00	22 (37.93))	20 (34.48))	0.35	0.78
	Yes	14 (13.08)	18 (16.82)			7 (12.07))	9 (15.52)		
Fracture	May 2A	29 (27.10)	28 (26.17)	1.79	0.24	22 (37.93)	20 (34.48)	0.07	> 0.05
	May 2B	19 (17.76)	31 (28.97)			7 (12.07)	9 (15.52)		
BMI		23.32 ± 3.29	22.47 ± 3.31	0.04	0.19	17 (29.31)	22.9 (19.14,25.15)	−0.156	0.88
Follow period (month)	33 (14,65)		41 (16,55)	−0.15	0.89	39 (15,65)	42 (22,53)	− 0.288	0.78

### Comparison of functional outcome and adverse events in group TB and group PF in 107 patients before PSM and in 58 patients after PSM

Before PSM, there was no significant difference between Group TBW and Group PF in terms of MEPS *(91.60 (86.7,94.75)* vs *92.2 (77.2,95.2), P = 0.56)*, flexion of elbow *(144(131,151)* vs *145 (133.5153.1), P = 0.36)*, forearm rotation *(146 (135,150)* vs *145 (135,150), P = 0.72)*, and RTW *(6 (3,9)* vs *5 (3,7), P = 0.13)*. A different cost was found between the TBW Group and the PF Group: the cost in PF Group was markedly higher than that in the TBW Group *(23,204.5 (18,400, 24,847)* vs *11,592 (9832, 17,102), P < 0.001))*. Before PSM, metalwork removal rate was higher in the TBW Group than in the PF Group *(46.43%* vs *16.64%, P < 0.001)* and other adverse events rate was higher in the PF Group than in the TBW Group *(12.5%* vs *8.93%, P < 0.001).* Total adverse events rate was higher in the TBW Group than in PF Group *(62.5%* vs *37.5%, P < 0.001)* (shown in Table 2).

**Table 2:** Comparison of functional outcome and adverse events in Group TB and Group PF in 107 patients before PSM and in 58 patients after PSM

		Group	Before PSM	P(χ2 /Z)	After PSM	P(χ2 /Z)
Functional outcomes	MEPS (QSD)	Tension band wiring	91.6 (86.7,94.75)	0.56(−0.59)	92 (86.8,94.3)	0.93(−0.09)
		Plate fixation	92.2 (77.2,95.2)		93.8 (87.9,95.5)	
	Elbow flexion (QSD)	Tension band wiring	144 (131,151)	0.36(−0.93)	144 (134,151)	0.62(−0.51)
		Plate fixation	145 (133.5153.1)		148 (135,153)	
	Forearm rotation (QSD)	Tension band wiring	146 (135,150)	0.72(−0.37)	145 (135,148.5)	0.5(−0.69)
		Plate fixation	145 (135,150)		148 (135,150)	
	RTW (QSD)	Tension band wiring	6 (3,9)	0.13(−1.53)	6 (3,9)	0.9(−0.14)
		Plate fixation	5 (3,7)		6 (3.5,9)	
Adverse events	Metalwork removal	Tension band wiring	26 (46.43)	< 0.001 (11.8)	19 (61.29)	0.03
		Plate fixation	11 (19.64)		5 (16.13)	
	Infection	Tension band wiring	2 (3.57)	> 0.05 (0.44)	1 (3.23)	–
		Plate fixation	2 (3.57)		1 (3.23)	
	Revision	Tension band wiring	2 (3.57)	0.59 (0.59)	1 (3.23)	–
		Plate fixation	1 (1.79)		0 (0)	
	Others	Tension band wiring	5 (8.93)	< 0.001 (18.44)	2 (6.45)	–
		Plate fixation	7 (12.5)		2 (6.45)	
	Total	Tension band wiring	35 (62.5)	< 0.001 (11.5)	23 (74.19)	0.001
		Plate fixation	21 (37.5)		8 (25.81)	
Cost (yuan)(QSD)		Tension band wiring	11,592 (9832,17,102)	< 0.001(−5.2)	11,943 (10,201,17,978)	< 0.001(−3.13)
		Plate fixation	23,204.5 (18,400,24,847)		23,519 (17,232,25,424.5)	

After PSM, there was no significant difference between Group TBW and Group PF in terms of MEPS *(92(86.8,94.3)* vs *93.8(87.9,95.5), P = 0.93)*, flexion of elbow *(144(134,151)* vs *148(135,153), P = 0.62)*, forearm rotation *(145(135,148.5)* vs *148(135,150), P = 0.5)*, and RTW *(6 (3,9)* vs *6(3.5,9), P = 0.9)*. After PSM, metalwork removal rate was higher in the TBW Group than in the PF Group *(61.29%* vs *16.13%, P = 0.03)* and total adverse events rate was higher in the TBW Group than in PF Group *(74.19%* vs *25.81%, P = 0.001)* (shown in Table 2).

### Univariate analysis of variables relating to treatment efficacy (MEPS> 90) and primary adverse events (metalwork removal) in 58 patients after PSM

As indicated by Table 3, a univariate analysis of 58 patients after propensity match scoring revealed that fracture type was significantly correlated with treatment efficacy (as indicated by MEPS> 90). The results of univariate analysis of metalwork removal were correlated with fracture type and age (Table 3).

**Table 3:** Univariate analysis of variables relating to treatment efficacy (MEPS> 90) and primary adverse events (metalwork removal) in 58 patients after PSM

		Treatment efficacy (MEPS> 90)		P(χ^2^/Z)	Primary adverse events (metalwork removal)		P(χ^2^/Z)
		Yes	No		No	Yes	
Sex	Male	18 (31.03)	14 (24.14)	1 (0.01)	17 (29.31)	15 (25.86)	0.27 (1.24)
	Female	15 (25.86)	11 (18.97)		10 (17.24)	16 (27.59)	
Occupation	No heavy labor	21 (36.21)	19 (32.76)	0.31 (1.02)	17 (29.31)	23 (39.66)	0.4 (0.85)
	Heavy labor	12 (20.69)	6 (10.34)		10 (17.24)	8 (13.79)	
Associated injury	None	14 (24.14)	15 (25.86)	0.71 (2.35)	9 (15.52)	20 (34.48)	0.12 (7.2)
	Ipsilateral shoulder joint injury	6 (10.34)	2 (3.45)		6 (10.34)	2 (3.45)	
	Ipsilateral wrist joints injury	4 (6.9)	3 (5.17)		3 (5.17)	4 (6.9)	
	Minor head injury	6 (10.34)	3 (5.17)		6 (10.34)	3 (5.17)	
	Others	3 (5.17)	2 (3.45)		3 (5.17)	2 (3.45)	
Mechanism of injury	Fall from standing height	13 (22.41)	11 (18.97)	0.95 (0.95)	13 (22.41)	11 (18.97)	0.9 (1.35)
	Fall from height	4 (6.9)	4 (6.9)		3 (5.17)	5 (8.62)	
	Motor vehicle collision	9 (15.52)	6 (10.34)		6 (10.34)	9 (15.52)	
	Sports	4 (6.9)	3 (5.17)		3 (5.17)	4 (6.9)	
	Fight/Assault	3 (5.17)	3 (5.17)		2 (3.45)	2 (3.45)	
Diabetes	No	31 (53.45)	25 (43.1)	0.50 (1.57)	25 (43.1)	31 (53.45)	0.21 (2.38)
	Yes	2 (3.45)	0 (0)		2 (3.45)	0 (0)	
Dominant hand	Left hand	0 (0)	0 (0)	–	0 (0)	0 (0)	–
	Right hand	33 (56.9)	25 (43.1)		27 (46.55)	31 (53.45)	
Side of injury	Left hand	19 (32.76)	17 (29.31)	0.59 (0.66)	15 (25.86)	21 (36.21)	0.42 (0.91)
	Right hand	14 (24.14)	8 (13.79)		12 (20.69)	10 (17.24)	
Alcohol consumption	< 21 units/wk	32 (55.17)	22 (37.93)	0.31 (1.78)	26 (44.83)	28 (48.28)	0.62 (0.8)
	> 21 units/wk	1 (1.72)	3 (5.17)		2 (3.45)	2 (3.45)	
Smoking	No	26 (44.83)	16 (27.59)	0.25 (1.56)	19 (32.76)	23 (39.66)	0.78 (0.11)
	Yes	7 (12.07)	9 (15.52)		8 (13.79)	8 (13.79)	
Group	TBW	15 (25.86)	14 (24.14)	0.6 (0.63)	6 (10.34)	23 (39.66)	< 0.001 (15.59)
	PF	18 (31.03)	11 (18.97)		21 (36.21)	8 (13.79)	
Fracture	Mayo 2A	23 (39.66)	10 (17.24)	0.03 (5.12)	18 (31.03)	15 (25.86)	0.19 (1.97)
	Mayo 2B	10 (17.24)	15 (25.86)		9 (15.52)	16 (27.59)	
Age		36 (25.5,51)		0.84(−0.2)	43 (33,66)	31 (22,42)	0.005(−2.79)
ASA grade		1 (1,1)		0.74(−0.34)	1 (1,1)	1 (1,2)	0.415(− 0.82)
BMI		22.46 ± 3.12		0.58 (0.31)	23.02 ± 0.57	22.2 ± 0.56	0.99 (1.02)

### Multivariate analysis of variables relating to treatment efficacy (MEPS> 90) and primary adverse events (metalwork removal) in 58 patients after PSM

A multivariate analysis revealed that fracture type was an independent factor that affected the efficacy *(regression coefficient = − 1.24 < 0, P = 0.03)*, indicating that fracture severity was inversely proportional to the treatment efficacy. The Mayo 2B efficacy rate was 29% of the Mayo 2A efficacy rate in terms of excellent MEPS (MEPS> 90). Furthermore, multivariate analysis demonstrated that group (treatment type) was an independent factor that affected metalwork removal of an olecranon fracture *(regression coefficient 2.38 > 0, OR = 10.77, P < 0.01)*, indicating that the risk of metalwork removal in Group TBW was 10.77 times higher than that in Group PF. Multivariate analysis demonstrated that age was an independent factor that affected metalwork removal *(regression coefficient = − 0.04 < 0, P = 0.03)*, indicating that for each additional year of age, the probability of metalwork removal is 96% of the probability of patients 1 year younger (Table 4). In other words, age is a protective factor against metalwork removal.

**Table 4:** Multivariate analysis of variables relating to treatment efficacy (MEPS> 90) and primary adverse events (metalwork removal) in 58 patients after PSM

	Variable		B	P	OR	95% confidence interval of OR	
						Low limit	Upper limit
Treatment efficacy	Fracture	Mayo 2B	−1.24	0.03	0.29	0.10	0.86
		Mayo 2A	0.00		1.00		
Primary adverse events	Age		−0.04	0.03	0.96	0.93	1.00
	TBW		2.38	< 0.01	10.77	2.91	39.80
	PF		0		1		

## Discussion

Although several novel treatments appeared promising, tension band wiring remains the gold standard within transverse simple olecranon fractures (Mayo 2A) [2, 4]. Plate fixation was preferred for comminuted olecranon fractures owing to better biomechanical compression across fractures. However, some scholars also recommended tension band wiring for comminuted olecranon fractures (Mayo 2B) owing to the comparable cost and shorter operation time [3, 7]. Previous studies have demonstrated the similar functional outcomes between TBW and PF. The treatment efficacy of the classical treatment methods (TBW and PF) for olecranon fractures is unclear [8]. Therefore, there is no agreement on which method provides the best outcome and should be recommended. The current study aimed to examine the differences between TBW and PF using propensity score matching analysis and aimed to explore which treatment method for displaced olecranon fractures is better. Propensity score matching was calculated for all 107 patients by logistic regression analysis for all 13 baseline variables and resulted in 29-matched cases. Though this is a retrospective study, the baseline variables between TBW group and PF group were balanced after PSM.

There was no difference in functional effects between Group TBW and Group PF (in terms of the MEPS, forearm arc, elbow flexion, and RTW), which was consistent with previous studies [3, 9, 10]. A multivariate analysis revealed that fracture type was an independent factor that affected the efficacy *(regression coefficient = − 1.24 < 0, P = 0.03)*, indicating that fracture severity was inversely proportional to the treatment efficacy. The Mayo 2B efficacy rate was 29% of the Mayo 2A efficacy rate in terms of excellent MEPS (MEPS> 90). As pointed out by previous studies, the implant irritation was higher in TBW group than that in PF group (12/13 vs 7/13) [11] and the more hardware removal in TBW group than that in PF group (10/58 patients vs 4/23, *P* = 0.0077) [12]. Hence, the metalwork removal rate was higher in the TBW group than in the PF group, which was confirmed again in the current study in both the original 107 patients recruited to participate in this study and in the 58 patients analyzed for propensity score matching. Given the position of internal fixation on the dorsal aspect ulna, the main perceived adverse events of olecranon fracture treatment are prominent hardware removal. Even if the tip portion of the cable and Kirschner wire were usually embedded in proximal ulna bone cortex by our chief doctors in the initial implant procedure, many patients chose a metalwork removal because of soft tissue stimulation of the metalwork. Metalwork removals were the major adverse events both in original 107 patients before PSM and in 58 patients after PSM. Metalwork removal was therefore adopted as the main perceived adverse events, and the potential factors affecting metalwork removal were investigated by binary logistic regression analysis. Interestingly, age and treatment options were two independent factors that affected metalwork removal from a statistical perspective. We have reasons to believe the reliability of the higher metalwork removal rate in TBW than PF because some experts have demonstrated that plate fixation provides significantly greater compression force than tension band wiring in the treatment of transverse fractures of the olecranon, both over the whole fracture and specifically at the articular side of the fracture [7, 12]. Another biomechanical advantage of this method is the better holding power of plate fixation and less soft tissue stimulation of the metalwork [7]. Although the cost of plate fixation in the current study was higher than that of tension band wiring *(23,519 yuan (17,232, 25,424.5yuan)* vs *11,943 yuan (10,201, 17978yuan))*, the overall expense may be higher for patients treated by TBW if the hospitalization and travel expenses incurred from the metalwork removal operation are considered [3, 13], which was similar to studies in other areas. For example, in a prospective study in England, overall median cost was higher in TBW group than that in PF group *(5546 £(2961–5654£)* vs *5174£ (3492–6828£))* [8] .Therefore, a second operation should carry considerable weight in the initial implant decision.

Multivariate analysis demonstrated that age was an independent factor that affected metalwork removal *(regression coefficient = − 0.04 < 0, P = 0.03)*, indicating that for each additional year of age, the probability of metalwork removal is 96% of the probability of patients 1 year younger. From a statistical perspective, age seems a protective factor against metalwork removal. However, age should not be regarded as an independent factor of metalwork removal: the false positive results may be related to underpowered effects and could possibly be resolved by increasing the sample size in future studies; the poor follow-up compliance of the elderly leads to a low positive record of subsequent adverse events. Conservative treatment or bone resection is still the first treatment option for low-demand elderly individuals with cancellous bone [14]. Plate fixation is still the preferred choice if internal fixation is decided for elderly individuals with olecranon fractures, owing to the more reliable biological stability in plate fixation [8].

A primary disadvantage of this study was the lack of blindness of both surgeons and patients to the allocated treatment method. It is argued that this is pragmatic in routine clinical practice, as people would always be aware of their proposed treatments. A further limitation to acknowledge is the fact that multiple surgeons with different experience levels were involved over the study period and people with concomitant injuries were involved in this study, which was also pragmatic and reflective of daily clinical practice [15]. Third, we could not exclude potentially subjective decisions to remove the metalwork. However, it is not routine to remove the metalwork unless the patient is symptomatic.

Based on similar functional outcomes between the two groups and more metalwork removals in the tension band wiring group, a second operation should carry considerable weight in the initial implant decision, especially for patients with Mayo 2B olecranon fracture.

## Conclusion

When initially discussing the surgical approach with patients, physicians should fully weigh the possibility that TBW may lead to a second surgery due to the higher risk of internal fixation removal and that TBW won’t yield better functional outcomes than PF .

## Data Availability

All data generated or analysed during this study are included in this article.

## Abbreviations

TBW: Tension band wiring
PF: Plate fixation
MEPS: Mayo Elbow Performance Score
ROM: Range of elbow motion
RFR: Range of forearm rotation
RTW: The time return to work
BMI: Body mass index
ASA: American Society of Anesthesiologists

## References

[1] Matar HE, Miller DJ, Duckett SP (2018). Metalwork prominence and operative interventions for treating olecranon fractures: systematic review of randomised controlled trials. J Long-Term Eff Med Implants.

[2] Matar HE, Ali AA, Buckley S, Garlick NI, Atkinson HD, Cochrane Bone, Joint and Muscle Trauma Group. Surgical interventions for treating fractures of the olecranon in adults. Cochrane Database Syst Rev. 2014. 10.1002/14651858.cd010144.pub2. 10.1002/14651858.CD010144.pub2 PMC6599821 25426876

[3] Duckworth AD, Clement ND, White TO (2017). Plate versus tension-band wire fixation for olecranon fractures: a prospective randomized trial. J Bone Joint Surg Am.

[4] Koziarz A, Woolnough T, Oitment C, Nath S, Johal H (2019). Surgical management for olecranon fractures in adults: a systematic review and meta-analysis. Orthopedics.

[5] Uhlmann M, Barg A, Valderrabano V (2014). Treatment of isolated fractures of the olecranon: percutaneous double-screw fixation versus conventional tension band wiring. Unfallchirurg.

[6] Di Francia R, Letissier H, Le Nen D (2019). Advantages of expulsion-proof pins in the treatment of olecranon fractures with tension band wiring: comparison with a control group. Orthopaed Traumatol Surg Res.

[7] Wilson J, Bajwa A, Kamath V (2011). Biomechanical comparison of interfragmentary compression in transverse fractures of the olecranon. J Bone Joint Surg (Br).

[8] Duckworth AD, Clement ND, McEachan JE (2017). Prospective randomised trial of non-operative versus operative management of olecranon fractures in the elderly. Bone Joint J.

[9] Amini MH, Azar FM, Wilson BR (2015). Comparison of outcomes and costs of tension-band and locking-plate Osteosynthesis in transverse olecranon fractures: a matched-cohort study. Am J Orthop (Belle Mead NJ).

[10] Hume MC, Wiss DA. Olecranon fractures: a clinical and radiographic comparison of tension band wiring and plate fixation. Clin Orthop Relat Res.1992;285:229–35. 1446443

[11] Schliemann B, Raschke MJ, Groene P (2014). Comparison of tension band wiring and precontoured locking compression plate fixation in Mayo type IIA olecranon fractures. Acta Orthop Belg.

[12] Tarallo L, Mugnai R, Adani R, Capra F, Zambianchi F, Catani F (2014). Simple and comminuted displaced olecranon fractures: a clinical comparison between tension band wiring and plate fixation techniques. Arch Orthop Trauma Surg.

[13] Powell AJ, Farhan-Alanie OM, McGraw IWW (2019). Tension band wiring versus locking plate fixation for simple, two-part Mayo 2A olecranon fractures: a comparison of post-operative outcomes, complications, reoperations and economics. Musculoskelet Surg.

[14] Symes M, Harris IA, Limbers J (2015). SOFIE: surgery for olecranon fractures in the elderly: a randomised controlled trial of operative versus non-operative treatment. BMC Musculoskelet Disord.

[15] Schwartz D, Lellouch J (2009). Explanatory and pragmatic attitudes in therapeutical trials. J Clin Epidemiol.

